# Artificial Intelligence in Glaucoma: Advances in Diagnosis, Progression Forecasting, and Surgical Outcome Prediction

**DOI:** 10.3390/ijms26104473

**Published:** 2025-05-08

**Authors:** Chiao-Hsin Lan, Ta-Hung Chiu, Wei-Ting Yen, Da-Wen Lu

**Affiliations:** 1 School of Medicine, National Defense Medical Center, Taipei 11490, Taiwan; sallylan410486@gmail.com; 2 Department of General Medicine, Tri-Service General Hospital, National Defense Medical Center, Taipei 114202, Taiwan; dennis963321@yahoo.com.tw; 3 Department of Ophthalmology, Tri-Service General Hospital, National Defense Medical Center, Taipei 114202, Taiwan

**Keywords:** glaucoma, artificial intelligence, optical coherence tomography, fundus photography, visual field, molecular biomarkers

## Abstract

Glaucoma is a leading cause of irreversible blindness, with challenges persisting in early diagnosis, disease progression, and surgical outcome prediction. Recent advances in artificial intelligence have enabled significant progress by extracting clinically relevant patterns from structural, functional, and molecular data. This review outlines the current applications of artificial intelligence in glaucoma care, including early detection using fundus photography and OCT and disease progression prediction using deep learning architectures such as convolutional neural networks, recurrent neural networks, transformer models, generative adversarial networks, and autoencoders. Surgical outcome forecasting has been enhanced through multimodal models that integrate electronic health records and imaging data. We also highlight emerging AI applications in omics analysis, including transcriptomics and metabolomics, for biomarker discovery and individualized risk stratification. Despite these advances, key challenges remain in interpretability, integration of heterogeneous data, and the lack of personalized surgical timing guidance. Future work should focus on transparent, generalizable, and multimodal AI models, supported by large, well-curated datasets, to advance precision medicine in glaucoma.

## 1. Introduction

Glaucoma is a common ophthalmic disease characterized by progressive optic nerve damage and visual field loss, making it one of the leading causes of irreversible blindness worldwide [1]. Conventional diagnostic techniques, including an intraocular pressure (IOP) measurement, optic nerve head evaluation, OCT imaging, and standard automated perimetry, require specialist interpretation and are subject to variability [2]. These diagnostic processes are resource-intensive and reliant on specialist expertise, yet even experienced clinicians show variability in detecting early glaucomatous changes [3].

Artificial intelligence offers a potential paradigm shift in glaucoma care. AI systems can learn complex patterns from large datasets and might detect subtle glaucomatous changes beyond the threshold of human observation. Over the past years, numerous studies have applied AI to ophthalmic diseases, including early detection of diabetic retinopathy, age-related macular degeneration (AMD), and glaucoma [4]. The primary clinical challenges in AI managing glaucoma care include early detection and diagnosis, accurate assessment of disease progression, and reliable prediction of surgical outcomes [5]. These factors directly influence patients’ ability to preserve their visual function and maintain their quality of life [5,6]. In recent years, the rapid advancements in medical imaging technologies and big data analysis have facilitated the integration of artificial intelligence into glaucoma multiple treatment decision-making [6]. By employing machine learning (ML) algorithms, AI can extract critical insights from large datasets of structural and functional tests, uncovering patterns in disease progression and identifying key risk factors. These capabilities provide clinicians with more objective and precise tools to inform clinical decisions [7]. Additionally, the growing incorporation of molecular-level data, such as transcriptomics and metabolomics, into AI frameworks enables novel biomarker discovery and enhances individualized risk stratification.

This review focuses on the latest applications of AI in various aspects of glaucoma care, including diagnosis, progression prediction, and surgical outcome forecasting from both imaging and molecular data, aiming to offer new perspectives and practical references for integrated glaucoma management in the future.

## 2. Artificial Intelligence in Glaucoma Early Detection and Diagnosis

### 2.1. AI and Color Fundus Photography in Glaucoma Early Detection and Diagnosis

Color fundus photography is widely used for glaucoma screening [8]. Deep learning (DL) models have been trained to detect characteristic glaucomatous changes, such as an increased cup-to-disc ratio, rim thinning, and nerve fiber layer defects [9,10,11]. Liu et al. (2019) developed a CNN-based system for detecting glaucomatous optic neuropathy (GON) using 274,413 fundus images, achieving an area under the receiver operating characteristic curve (AUROC) of 0.996, with a sensitivity of 96.2% and specificity of 97.7% [10]. Similarly, Phene et al. (2019) also evaluated a DL model for detecting referable glaucomatous optic neuropathy (GON) and optic nerve head (ONH) features, demonstrating robust performance with an area under the curve (AUC) of 0.945, 0.855, and 0.881 across three datasets [11]. Notably, the model achieved higher sensitivity than 7 out of 10 graders, including two of three glaucoma specialists, with comparable specificity, suggesting its potential utility as a reliable screening tool [11]. 

In addition to the detection and diagnosis of existing glaucomatous changes, deep learning models have also shown potential in predicting the development of glaucoma before clinical onset [12,13,14,15]. Such predictive models, trained on large datasets of fundus photographs, have demonstrated reasonable accuracy, even years before detectable disease manifestations [12]. One deep learning model achieved an AUC of 0.77 for predictions made 4–7 years prior to glaucoma onset, an accuracy of 0.88 for predictions 1–3 years prior, and an accuracy of 0.95 after glaucoma onset [12]. These findings highlight the possibility of using AI not only for diagnosing established glaucoma but also for identifying individuals at high risk of developing glaucoma, thereby enabling earlier interventions and closer monitoring. Moreover, deep learning models based on color fundus photography, such as the Machine-to-Machine (M2M) model, have demonstrated the ability to predict glaucomatous conversion by estimating the RNFL thickness from fundus photographs [16]. In a study of 1072 eyes from 827 glaucoma suspects, the M2M model achieved hazard ratios of 1.56 per 10 μm lower baseline thickness and 1.99 per 1 μm/year faster decline, showing a comparable performance to OCT measurements and highlighting its potential as a low-cost, accessible screening tool [16]. Recent advancements have addressed the challenge of irregularly sampled images in glaucoma progression prediction. A transformer-based model named GLIM-Net incorporated time positional encoding and time-sensitive multi-head self-attention modules, achieving superior accuracy in predicting glaucoma progression from irregularly sampled fundus images [15].

Additionally, other studies have reported promising results using fundus images for AI-based glaucoma screening, achieving high sensitivity and specificity across various datasets [17,18,19,20,21,22]. Overall, the application of AI models based on color fundus photography significantly enhances glaucoma screening and early detection, providing reliable diagnostic accuracy and the potential for earlier intervention and monitoring in clinical practice. Table 1 summarizes the key representative studies discussed above.

### 2.2. AI and OCT in Glaucoma Early Detection and Diagnosis

Optical coherence tomography (OCT) is a critical imaging modality for detecting and diagnosing glaucomatous structural changes [23,24]. Numerous deep learning (DL) models have been developed to analyze various OCT imaging formats, including standard OCT reports, cross-sectional scans, 3D volumetric imaging, anterior segment OCT, and OCT angiography (OCTA) [25,26,27,28]. The unique structural details captured by each modality enhance the performance of convolutional neural networks (CNNs) in distinguishing glaucomatous eyes from healthy controls and estimating retinal nerve fiber layer (RNFL) thickness [29].

Several studies have demonstrated excellent diagnostic accuracy with DL models applied to OCT data, particularly for early-stage glaucoma detection. Braeu et al. (2023) developed PointNet and dynamic graph convolutional neural network (DGCNN) models, achieving AUCs exceeding 0.95 and 0.97, respectively, using optic nerve head (ONH) scans [30]. They identified the inferior and superior quadrants of the neuroretinal rim as critical regions for glaucoma diagnosis [30]. Similarly, Wu et al. (2021) compared multiple machine learning models, including conditional inference trees (CIT), logistic model tree (LMT), C5.0 decision tree, random forest (RF), and extreme gradient boosting (XGBoost), and found that random forest (RF) was the best performing model [31]. They also found that ganglion cell layer measurements were the most predictive of early glaucoma, whereas retinal nerve fiber layer thickness metrics were more indicative of advanced disease [31].

While convolutional neural networks (CNNs) have demonstrated strong performance in spatial feature extraction, recurrent neural networks (RNNs), such as long short-term memory (LSTM) and gated recurrent unit (GRU) models, offer advantages in capturing sequential dependencies within imaging data. These models have shown potential in analyzing structural sequences derived from volumetric OCT scans. In this context, Ashtari-Majlan et al. (2025) proposed a spatial-aware transformer–gated recurrent unit (GRU) framework that leveraged three-dimensional optical coherence tomography (3D OCT) imaging for glaucoma diagnosis [32]. Their model integrated a Vision Transformer to extract rich slice-wise features from volumetric OCT data and a bidirectional GRU to model spatial dependencies across B-scans [32]. This architecture achieved an AUC of 94.20% and an F1 score of 93.01%, demonstrating that incorporating sequential structural information within 3D OCT volumes can significantly enhance diagnostic performance [32].

In another study, Li et al. (2023) trained models using retinal nerve fiber layer thickness (RNFLT) data corrected for anatomical factors and demonstrated improved diagnostic performance in Caucasian populations compared to models using uncorrected data [14]. The study involved 514 Asians for model training and 356 Asians for testing, highlighting the importance of considering ethnic differences in AI applications [14]. These studies collectively illustrate that DL models leveraging OCT data offer exceptional diagnostic accuracy for glaucoma detection, highlighting the importance of proper data compensation and cross-ethnic validation to ensure broad applicability and reliability.

Furthermore, optical coherence tomographic angiography (OCTA) is a non-invasive imaging technique that provides three-dimensional, high-resolution vascular images [33]. Recently, OCTA has demonstrated significant potential in AI-based glaucoma detection [34]. In a study by De Jesus et al. (2020), an automated classification algorithm was developed using OCTA scans to assess glaucomatous vascular damage, achieving AUC values of 0.89 for support vector machine (SVM), 0.86 for random forest (RF), and 0.85 for gradient boosting (xGB) in diagnosing glaucoma [35]. In a related study, Bowd et al. (2022) demonstrated that deep learning analysis using convolutional neural networks (CNN) of en face vessel density images significantly improved the classification of healthy and glaucomatous eyes, achieving an adjusted area under the precision–recall curve of 0.97, which outperformed gradient boosting classifier models based on OCTA and OCT measurements [36]. These findings further highlight the potential of AI-based OCTA analysis in enhancing glaucoma detection and staging, with deep learning models providing valuable improvements over traditional feature-based methods.

Additionally, recent evidence indicates that deep learning models utilizing macular OCTA images exhibit excellent diagnostic performance even in glaucomatous eyes with high myopia [37]. Lee et al. (2023) developed a deep learning model to evaluate the diagnostic value of macular microvasculature using swept-source OCTA in highly myopic glaucoma [37]. The model achieved an AUC of 0.946 with superficial capillary plexus images, a performance comparable to macular OCT images and significantly superior to deep capillary plexus images. These findings suggest that macular OCTA microvasculature may serve as a potential biomarker for glaucoma diagnosis in eyes with high myopia [37].

In summary, the integration of AI and OCT imaging has significantly enhanced the accuracy and efficacy of glaucoma detection and diagnosis. The consistent performance of DL models across diverse OCT modalities and ethnic populations underscores their potential for widespread clinical application, emphasizing the need for continued validation and optimization to maximize their diagnostic performance. As shown in Table 2, the key representative studies on AI-driven OCT-based glaucoma detection and diagnosis are summarized below.

### 2.3. AI and Molecular Data Analysis in Glaucoma Diagnosis and Detection

In addition to the imaging-based approaches, artificial intelligence has demonstrated increasing utility in the analysis of molecular and omics data to facilitate glaucoma diagnosis and risk stratification. Through the integration of high-throughput technologies—such as metabolomics, transcriptomics, and genomics—AI enables the identification of novel biomarkers and genetic drivers underlying glaucoma pathogenesis, offering new directions for both early detection and individualized management. AI has also been applied in transcriptomic biomarker discovery. Dai et al. (2022) analyzed publicly available RNA sequencing datasets (GSE9944 and GSE2378) and employed logistic regression, random forest, and least absolute shrinkage and selection operator (LASSO) algorithms to construct diagnostic models and identify three candidate genes (ENO2, NAMPT, and ADH1C) relevant to glaucoma [38]. Notably, ENO2 was found to be significantly downregulated in glaucomatous tissues and negatively correlated with the expression of NAMPT and ADH1C [38]. Subsequent molecular docking analyses revealed that ENO2 may serve as a viable therapeutic target, thus illustrating a complete AI-enabled pipeline from gene prioritization to drug discovery [38].

On the genomics front, Han et al. (2021) applied deep learning to annotate the vertical cup-to-disc ratio (VCDR) and vertical disc diameter (VDD) across more than 280,000 fundus images obtained from the UK Biobank and the Canadian Longitudinal Study on Aging [39]. This large-scale AI-derived phenotyping enabled a high-powered genome-wide association study (GWAS), which identified over 200 loci implicated in optic nerve head morphology, many of which were previously linked to glaucoma susceptibility [39]. Similarly, Alipanahi et al. (2021) demonstrated that ML-based phenotyping of fundus images could improve GWAS efficiency, identifying 93 novel loci associated with VCDR and enhancing both heritability estimates and polygenic risk prediction for primary open-angle glaucoma [40].

More recently, Sergouniotis et al. (2024) introduced an unsupervised autoencoder-based framework to extract latent structural features from optical coherence tomography (OCT) images of over 31,000 UK Biobank participants [41]. The resulting 64-dimensional embeddings were used in a GWAS that identified 118 significant loci, including several implicated in ophthalmic and neurodegenerative conditions [41]. These AI-derived features also demonstrated predictive value for both glaucoma and systemic diseases such as cardiovascular disorders, thereby highlighting the broader translational potential of self-supervised image phenotyping [41].

Collectively, these studies underscore the transformative role of AI in molecular-level glaucoma research. By enabling scalable, reproducible, and high-resolution analyses of omics and image-derived data, AI facilitates comprehensive biomarker discovery, improves genetic mapping, and supports integrative diagnostic strategies. Future investigations combining multi-omics datasets, longitudinal imaging, and clinical outcomes will be critical to advancing precision medicine approaches in glaucoma management. Table 3 provides a concise overview of the representative studies that apply AI to molecular and omics data for glaucoma diagnosis and detection.

## 3. AI in Predicting Glaucoma Progression

Accurate prediction of glaucoma progression is essential for effective clinical management, facilitating early intervention and individualized treatment strategies [42]. Optical coherence tomography and visual field assessments are commonly utilized to monitor glaucoma progression severity [43,44]. Recent advancements in machine learning models have demonstrated enhanced predictive accuracy in forecasting OCT and visual field deterioration by integrating structural and functional assessment data [45].

### 3.1. AI and OCT in Glaucoma Progression

Advancements in deep learning have markedly improved glaucoma progression assessment through OCT imaging, notably employing recurrent neural networks (RNNs), gated transformer networks (GTNs), generative adversarial networks (GANs), and autoencoders. These approaches have demonstrated potential in enhancing diagnostic accuracy, monitoring disease progression, and providing novel insights into glaucoma pathophysiology.

Recurrent neural networks utilize temporal sequences of OCT scans to model longitudinal changes [46]. Among various RNN architectures, long short-term memory (LSTM) networks are particularly effective in capturing long-range temporal dependencies by incorporating memory cells and gating mechanisms, thereby addressing the limitations of traditional RNNs such as vanishing gradients [46]. Mandal et al. (2024) used a convolutional long short-term memory (LSTM) network to distinguish true glaucomatous thinning from age-related changes in serial peripapillary OCT scans, achieving a significantly higher hit rate (0.498) compared to conventional trend-based regression (0.284), demonstrating its capability to detect glaucoma progression [46].

Building upon advances in sequence modeling, gated transformer networks (GTNs) utilize self-attention mechanisms to effectively capture temporal dynamics within longitudinal OCT datasets [47]. The GTN architecture offers several distinct advantages over traditional recurrent neural network (RNN)-based approaches, including improved computational efficiency through parallelization, superior capacity to capture long-range dependencies, and greater flexibility in modeling complex temporal relationships, making it particularly suited for high-dimensional ophthalmic imaging data [47]. Hou et al. (2023) introduced a GTN-based model trained on longitudinal OCT scans to predict glaucoma progression, employing a composite metric termed “Majority of 6” (M6) that defined progression by consensus among six independent evaluation criteria [47]. Under this rigorous M6 criterion, the GTN model achieved an impressive AUC of 0.97, significantly surpassing conventional trend-based analyses [47]. Notably, the model demonstrated heightened sensitivity to early glaucomatous progression through precise detection of subtle changes in retinal nerve fibre layer (RNFL) thickness [47]. These findings underscore the substantial potential of transformer-based deep learning architectures in enhancing predictive accuracy and providing robust support for clinical decision-making in glaucoma management [47].

Generative adversarial networks (GANs) have recently attracted significant interest in simulating structural progression in glaucoma [48,49,50]. Hassan et al. (2020) developed a conditional GAN model capable of generating follow-up optical coherence tomography (OCT) B-scans based on two or three prior scans, achieving retinal thickness predictions that closely matched actual six-month follow-up images [48]. Similarly, Lazaridis et al. (2021) introduced a probabilistic ensemble of cyclical GANs (CycleGANs) to enhance statistical power in glaucoma clinical trials using time-domain OCT (TDOCT) [49]. Their method converted low signal-to-noise ratio TDOCT images into synthesized high-resolution spectral-domain OCT (SD-OCT) images, subsequently performing retinal nerve fibre layer segmentation via Bayesian fusion of multiple GAN-generated images [49]. This approach significantly improved the differentiation between treatment arms, thereby increasing the statistical efficacy comparable to visual field measurements [49]. More recently, Hussain et al. (2023) utilized GAN-generated synthetic OCT images to enhance the predictive accuracy of a multimodal deep learning model combining convolutional neural networks (CNNs) and long short-term memory (LSTM) networks [50]. Their framework integrated OCT images, visual field data, and clinical demographics to predict visual field deterioration up to 12 months in advance [50]. The GAN-generated images significantly improved the model’s predictive capability, demonstrating superior accuracy compared to models using structural or functional data independently [50]. Collectively, these findings highlight the potential of GAN-based methodologies in advancing the prediction and monitoring of glaucoma progression using OCT imaging.

Autoencoders, designed for dimensionality reduction and feature extraction, have been applied to OCT imaging to enhance the assessment of glaucoma progression. Bowd et al. (2021) employed unsupervised deep-learning autoencoders (DL-AEs) to generate individualized, eye-specific regions of interest (ROIs) from segmented retinal nerve fiber layer (RNFL) data in 3D OCT images [51]. Compared to conventional global circumpapillary RNFL (cpRNFL) thickness measurements, the DL-AE-derived ROIs demonstrated significantly higher sensitivity in detecting structural glaucomatous progression (90% vs. 63%), while maintaining comparable specificity (92% vs. 93%) [51]. Moreover, the mean rate of RNFL thinning within the DL-AE ROIs was significantly faster than in cpRNFL measurements, indicating enhanced ability to detect subtle longitudinal changes [51]. These findings support the clinical utility of individualized ROI-based analysis using autoencoders to improve the detection and monitoring of glaucomatous progression.

In summary, deep learning methodologies involving RNNs, GTNs, GANs, and autoencoders have demonstrated considerable promise for enhancing OCT-based glaucoma progression analysis, offering improved diagnostic precision and novel evaluation strategies for structural disease changes. Table 4 synthesizes the key studies applying AI to OCT-based glaucoma progression analysis, detailing model architectures, datasets, and primary performance metrics.

### 3.2. AI and Visual Field in Glaucoma Progression

Recent advancements in artificial intelligence have significantly enhanced the evaluation of visual field (VF) progression in glaucoma through the integration of structural and functional data [52]. Mohammadzadeh et al. (2024) utilized a Siamese Neural Network architecture based on ResNet-152 with transfer learning pretrained on ImageNet, to predict VF progression by analyzing optic disc photographs (ODP) and retinal nerve fiber layer (RNFL) thickness measurements from 3079 eyes [52]. Their findings revealed that the combined baseline ODP and RNFL thickness data produced an AUC of 0.813 [52]. The addition of the second sequential ODP improved the AUC to 0.860, while incorporating the third sequential ODP further elevated the AUC to 0.894. Importantly, the model demonstrated an AUC of 0.911 in identifying rapid glaucoma progression, defined as a mean deviation deterioration rate exceeding 1.0 dB per year, highlighting its potential for early detection of patients at high risk. The synergistic application of quantitative RNFL parameters and qualitative morphological features from ODP images represents a robust approach for accurately assessing and forecasting glaucoma progression, underscoring its potential clinical applicability [52].

Building on this, Mohammadzadeh et al. (2024) applied a twin-neural network with a ResNet-50 backbone to analyze baseline and follow-up optic disc photographs (ODPs) from 3919 eyes, among which 19% exhibited visual field progression, defined as a statistically significant negative mean deviation slope across two consecutive visits and the final visit [53]. The model achieved an area under the receiver operating characteristic curve of 0.862 for predicting any VF progression, and an AUC of 0.926 for rapid progression, defined as an MD reduction exceeding 1.0 dB/year [53]. The model was trained using longitudinal pairs of ODPs acquired at least two years apart and employed extensive image augmentations and transfer learning strategies to enhance generalizability. Notably, saliency map analyses using XRAI indicated that the model focused on clinically relevant structural features such as neuroretinal rim thinning and peripapillary changes, which may precede detectable functional decline. These findings underscore the clinical utility of ODP-based deep learning models in enabling earlier identification of glaucomatous progression, especially in fast-progressing eyes, and highlight their potential applicability in resource-limited settings where advanced imaging tools may be unavailable [53].

Another notable application of artificial intelligence in the context of glaucoma progression monitoring is the development of visualization tools [54]. Yousefi et al. (2020) proposed a glaucoma dashboard that leverages dimensionality reduction techniques to categorize visual field patterns into 32 distinct clusters, each representing different degrees and configurations of glaucomatous damage [54]. By analyzing a large dataset comprising more than 13,000 visual field examinations, the dashboard demonstrated a specificity of 94% for identifying stable fields and a sensitivity of 77% for detecting progression [54]. This cluster-based visualization approach enables intuitive tracking of VF changes over time, offering clinicians a user-friendly platform for real-time assessment of disease trajectories and enhancing decision-making in clinical settings [54].

The integration of spatiotemporal visual field data with clinical parameters has further improved the ability of artificial intelligence models to assess glaucoma progression. Dixit et al. (2021) developed a convolutional long short-term memory neural network trained on longitudinal visual field sequences, alongside clinical variables, including cup-to-disc ratio, central corneal thickness, and intraocular pressure [55]. The model demonstrated an AUC of 0.79–0.82 when trained solely on VF data, which increased to 0.89–0.93 upon incorporating clinical data, indicating a statistically significant improvement [55]. Notably, the model achieved 91% to 93% accuracy in identifying progression across three conventional progression algorithms—the VF index slope, mean deviation slope, and pointwise linear regression—given four sequential VF results. These findings highlight the value of combining structural and functional clinical information with temporal VF trends, supporting the use of multidimensional data fusion for more accurate and clinically relevant prediction of glaucoma progression [55].

Beyond convolutional architectures, a variety of machine learning and deep learning models have been applied to longitudinal VF data for glaucoma progression prediction [56]. Saeedi et al. (2021) analyzed over 90,000 VF tests from 13,156 eyes using six machine learning classifiers, including logistic regression, random forest, extreme gradient boosting, support vector classifier, convolutional neural network, and fully connected neural network, to classify progression based on six conventional VF progression algorithms [56]. These models achieved accuracies ranging from 87% to 91%, with sensitivities between 0.83 and 0.88, and specificities between 0.92 and 0.96. Notably, while traditional algorithms exhibited significant class bias in borderline cases, the machine learning classifiers demonstrated balanced decision-making, suggesting their potential for more consistent and reliable assessment of VF progression [56].

Similarly, Shuldiner et al. (2021) trained multiple machine learning classifiers on initial visual field data from over 22,000 eyes to predict rapid glaucoma progression, defined as a mean deviation decline exceeding 1.0 dB per year [57]. Among the tested models, the support vector machine achieved the highest performance, with an AUC of 0.72. Notably, the inclusion of a second VF did not significantly improve model performance, indicating that prognostically meaningful information is already embedded in the first VF [57]. Variables most predictive of rapid progression included older age and higher pattern standard deviation, suggesting that early VF abnormalities can serve as valuable markers for identifying high-risk patients [57].

More recently, Sabharwal et al. (2023) introduced a deep learning model incorporating both spatial and temporal features of visual field sequences, trained on labels derived from a consensus of six conventional progression algorithms [58]. Using a dataset of 8705 eyes from over 5000 patients, the model achieved a high AUC of 0.94 for identifying VF worsening in the held-out test set [58]. Notably, even when the most recent six VF tests were excluded—simulating limited follow-up—the model still maintained robust performance with an AUC of 0.78. In contrast, clinician assessments based on electronic health record documentation achieved an AUC of only 0.64, highlighting the model’s superior consistency and potential utility in routine clinical decision-making [58]. These findings underscore the value of consensus-based labeling and spatiotemporal modeling for reliable VF progression detection, particularly in cases with shorter follow-up or subjective clinical ambiguity [58].

In addition, transformer-based deep learning models have recently been proposed to forecast region-specific visual field progression. Chen et al. (2024) developed a multi-label transformer-based network (MTN) trained on longitudinal VF data to predict progression within six anatomically defined VF regions mapped to the optic disc [59]. Using data from 2430 eyes with at least five VF tests, the model achieved macro-average AUCs exceeding 0.88 for detecting focal progression [59]. With six VF inputs, the model demonstrated more stable and superior performance, including an AUC of 0.848 when forecasting deterioration across eight future tests. In eyes with severe baseline loss (MD ≤ −12 dB), the MTN still maintained strong discriminative ability in most regions (AUC ≥ 0.86, sensitivity 1.0, specificity ≥ 0.70), underscoring its potential in guiding personalized glaucoma management even in advanced disease [59].

In summary, these advances collectively illustrate the increasing capability and flexibility of AI in accurately predicting and monitoring glaucoma progression using visual field data. The integration of structural and functional data, advanced visualization methods, and novel modeling techniques exemplifies AI’s significant potential to enhance early detection, facilitate personalized management, and improve patient outcomes. As shown in Table 5, the principal studies applying AI to visual field data for glaucoma progression prediction are summarized below.

### 3.3. AI and Molecular Data Analysis in Glaucoma Progression

Artificial intelligence has become a powerful tool for analyzing molecular data, enabling the identification of biomarkers from complex omics datasets such as metabolomics, proteomics, and genomics. These approaches support the development of non-invasive and data-driven strategies for glaucoma diagnosis and monitoring. Li et al. (2024) applied machine learning algorithms to targeted metabolomics data and identified androstenedione as a key serum biomarker for primary angle-closure glaucoma [60]. In a multicenter study involving 616 participants, androstenedione demonstrated strong diagnostic performance, with an area under the curve reaching up to 1.0 in discovery sets and 0.86 to 0.87 in validation cohorts [60]. Furthermore, higher baseline levels of androstenedione were associated with more rapid visual field progression over a two-year follow-up. These findings illustrate the potential of AI-integrated metabolomics to enhance both early detection and prognostication of glaucoma, paving the way for precision medicine in clinical ophthalmology [60].

Beyond molecular biomarkers, artificial intelligence frameworks are increasingly being developed to integrate diverse data modalities, including ocular imaging, functional tests, and multi-omics profiles, to improve individualized risk stratification in glaucoma [61]. Recent studies have proposed multimodal deep learning systems that simultaneously analyze optical coherence tomography scans, visual field series, clinical parameters, and patient demographics to forecast disease progression [61]. These integrative models aim to democratize glaucoma management by enabling earlier identification of rapid progressors and guiding timely, personalized interventions [61]. In parallel, the incorporation of genomic data into such frameworks is gaining traction, reflecting the evolving consensus that a combination of genetic risk factors, molecular signatures, and ocular phenotypes will offer the most robust predictive power [61]. Collectively, these multimodal AI approaches represent a critical frontier in glaucoma research, with the potential to refine prognostic accuracy and support precision medicine initiatives in clinical ophthalmology.

## 4. Artificial Intelligence in Glaucoma Surgical Outcome Prediction

In recent years, the rising prevalence of glaucoma has driven efforts to evaluate surgical outcomes and develop personalized treatment plans using artificial intelligence [62]. Artificial intelligence has emerged as a valuable tool in predicting surgical outcomes for glaucoma, aiming to enhance postoperative management and improve patient care through personalized treatment strategies. Several studies have demonstrated the potential of AI in predicting surgical success rates and failure risks by analyzing diverse patient data inputs [63,64].

Banna et al. (2022) investigated 230 trabeculectomy cases using machine learning models, including random forest (RF), support vector machines (SVM), and artificial neural networks (ANN) [63]. Their findings revealed that the RF model achieved moderate predictive performance with an accuracy of 0.67 and an AUC of 0.68 using demographic and ocular data [63]. Notably, when systemic features such as cardiovascular history and medication records were incorporated, the model’s accuracy improved to 0.68, and the AUC increased to 0.74, highlighting the importance of multidimensional data integration [63].

Building upon these findings, Barry et al. (2024) examined 2398 surgical cases involving various techniques, including trabeculectomy and Minimally Invasive Glaucoma Surgeries (MIGS) [64]. Utilizing models such as RF, gradient boosting, multilayer perceptron (MLP), and deep neural networks (DNN), they identified that gradient boosting achieved the highest performance in predicting failure in intraocular pressure control, with an AUC of 0.855. This study emphasized the value of integrating electronic health records (EHR), clinical variables, and medical imaging to enhance predictive performance, particularly in complex surgical scenarios [64].

Lin et al. (2024) advanced this area by developing a multimodal deep learning model integrating free-text operative notes with structured EHR data to improve prediction accuracy for multiclass glaucoma surgical outcomes [65]. This approach achieved a macro AUROC of 0.750 and an F1 score of 0.583, surpassing the structured data-only models (AUROC of 0.712 and F1 score of 0.486). Additionally, the multimodal model demonstrated superior recall for hypotony-related surgical failures and higher precision for predicting successful outcomes, underscoring the importance of incorporating intraoperative information for accurate predictive modeling [65].

Furthermore, Agnifili et al. (2023) applied a classification tree (CT) algorithm to predict filtration surgery (FS) outcomes in 102 glaucomatous patients, incorporating ocular surface clinical tests (OSCTs), surgical site-related biometric parameters (SSPs), and conjunctival vascularization [66]. Their findings demonstrated that conjunctival stromal thickness (CST) and reflectivity (SCR), along with age, were the most influential predictors, with CST serving as the most critical factor. The ROC curve for their classifier showed a good accuracy (AUC of 0.784), and the study concluded that thicker and hyper-reflective conjunctival stroma and younger age were associated with an increased risk of FS failure, further supporting the utility of multimodal data integration [66].

In addition, Mastropasqua et al. (2023) introduced a deep learning model designed to classify filtration bleb (FB) functionality post-trabeculectomy using slit-lamp images [67]. The model was trained and validated on 119 FB images, achieving an accuracy of 74%, sensitivity of 74%, specificity of 87%, and an AUC of 0.8. The Kappa coefficient of 0.58 and statistical significance underscore the model’s effectiveness in distinguishing functioning from failed FBs, particularly in situations where adjunctive clinical data is unavailable [67].

Taken together, these studies underscore the expanding potential of artificial intelligence to support surgical outcome prediction in glaucoma. Through the application of deep learning and ensemble learning models, and by incorporating a wide spectrum of data, including structured EHRs, free-text operative notes, clinical measurements, and medical imaging, AI systems offer a powerful framework for risk stratification, postoperative monitoring, and personalized care planning in glaucoma surgery. Table 6 synthesizes the principal studies employing AI for glaucoma surgical outcome prediction, detailing their methodologies, data inputs, and key performance metrics.

## 5. Discussion and Future Directions

Artificial intelligence has increasingly contributed to glaucoma management, with promising developments in early detection, monitoring of disease progression, and prediction of surgical outcomes. Nevertheless, several important challenges remain before these technologies can be fully integrated into clinical practice. Issues such as model interpretability, data heterogeneity, lack of personalized surgical timing guidance, and limited use of molecular-level data continue to restrict their broader applicability.

One of the most commonly cited concerns involves the interpretability of AI models. Many current algorithms, particularly deep learning frameworks, generate outputs without offering a transparent rationale behind their predictions. In clinical settings, especially when guiding high-stakes decisions such as glaucoma surgery, this “black box” nature may limit physician confidence and adoption [68]. Moving forward, greater emphasis on model explainability is warranted. Techniques that highlight relevant features or visualize decision pathways could help bridge the gap between algorithmic prediction and clinical reasoning.

Another important challenge lies in the integration of multimodal data. While structured information such as OCT metrics, intraocular pressure, and demographic variables is frequently used in AI models, the incorporation of unstructured data, such as free-text notes, imaging annotations, and longitudinal narrative summaries, remains underdeveloped. Advances in natural language processing and multimodal learning architectures may allow for more comprehensive patient modeling, capturing subtleties not reflected in structured variables alone.

In glaucoma surgical decision-making, most artificial intelligence models aim to predict postoperative outcomes, but few provide guidance on the optimal timing of intervention. This is particularly important for patients experiencing rapid functional or structural decline. Future models should integrate longitudinal indicators, such as the rate of visual field loss or retinal nerve fiber layer thinning, to support more informed and individualized surgical decisions. 

In addition, another area of growing interest is the use of AI to analyze molecular-level data for predicting glaucoma progression. Despite advances in genomics, transcriptomics, epigenetics, proteomics, and metabolomics, there remains a paucity of studies applying machine learning to these data types with the specific goal of forecasting disease progression. Most existing studies focus on identifying potential biomarkers using conventional statistical methods. The development of models that incorporate molecular features, such as gene expression profiles, methylation patterns, or metabolomic signatures, could improve risk stratification, particularly when combined with imaging and clinical data. For example, metabolic endotypes or epigenetic aging markers may offer insights into progression risk that are not readily apparent from structural imaging alone.

Although these omics-based approaches are still in their early stages, the integration of molecular data into AI models may enable more refined predictions of disease behavior, uncover mechanisms of fast progression, and potentially identify subgroups of patients who could benefit from tailored monitoring or early intervention. Combining molecular, clinical, and imaging data into unified frameworks represents a logical and potentially impactful next step in glaucoma precision medicine.

In summary, while AI has demonstrated substantial value in several aspects of glaucoma care, there remains significant room for further development, particularly in areas involving interpretability, multimodal data integration, surgical timing guidance, and the incorporation of molecular-level information. Addressing these gaps will be essential for translating AI tools into reliable and widely applicable support systems for clinical decision-making in glaucoma management.

## 6. Conclusions

Artificial intelligence is playing an increasingly important role in glaucoma care, with applications in early diagnosis, monitoring of disease progression, and prediction of surgical outcomes. The integration of clinical data, imaging, and operative records has improved predictive accuracy and enabled more personalized decision-making. Moreover, incorporating molecular-level information such as transcriptomic and metabolomic profiles into AI models offers new opportunities for biomarker discovery and individualized risk stratification. To ensure broader clinical adoption, future research should focus on developing explainable and generalizable AI models, integrating techniques such as attention-based visualization and model interpretability frameworks. Alongside these efforts, the use of federated learning for privacy-preserving model training and prospective multicenter validation studies will be critical for safe and ethical implementation in clinical settings. Establishing large, diverse, and well-annotated datasets through international collaboration will support these efforts. Ultimately, AI-driven tools are expected to refine precision medicine in glaucoma and improve long-term patient outcomes.

## Figures and Tables

**Table 1:** Summary of AI models for glaucoma detection and early prediction using color fundus photography.

Study	Year	Study Design	Data Type	Data Size	AI Type	Task	Performance
Liu et al. [10]	2019	Cross-sectional	Color fundus photographs.	274,413 images.	GD-CNN (ResNet-based).	Detects glaucomatous optic neuropathy (GON).	AUROC: 0.996; Sensitivity: 96.2%; Specificity: 97.7%.
Phene et al. [11]	2019	Retrospective	Color fundus photographs.	86,618 images.	Deep convolutional neural network with the Inception-v3 architecture.	Predicts referable GON and optic nerve head (ONH) features.	AUROC: 0.945.
Thakur et al. [12]	2020	Prospective longitudinal study	Color fundus photographs.	66,721 images (from 1636 patients).	Convolutional neural network (MobileNetV2).	1. Glaucoma diagnosis 2. Predict conversion 1–3 yrs prior 3. Predict conversion 4–7 yrs prior.	1. Diagnosis: AUC 0.945; 2. Prediction 1–3 years prior onset: AUC 0.88; 3. Prediction 4–7 years prior onset: AUC 0.77.
Hu et al. [15]	2023	Retrospective evaluation of existing sequential-image datasets	Sequential fundus images.	3671 images (from 405 eyes).	Transformer (GLIM-Net).	Glaucoma detection and progression forecast.	Accuracy 89.5%; Sensitivity 87.6%; Specificity 89.6%; AUC 93.6%.
Lee et al. [16]	2021	Retrospective cohort study	Color fundus photographs, standard automated perimetry (SAP).	1072 eyes of 827 glaucoma-suspect patients.	Convolutional neural network (M2M model); joint longitudinal survival model.	Predict future development of glaucomatous visual field defects.	Survival Prediction Results: HR for conversion risk: 1.56 per 10 μm lower baseline predicted RNFL thickness. HR for conversion risk: 1.99 per 1 μm/year faster loss in predicted RNFL thickness.

**Table 2:** Summary of key representative studies on AI and OCT in glaucoma detection and early diagnosis.

Study	Year	Study Design	Data Type	Data Size	AI Type	Task	Performance
Braeu et al. [30]	2023	Retrospective (comparison and evaluation of deep learning diagnostic algorithms)	OCT scans of the optic nerve head (ONH).	4506 OCT scans (2247 non-glaucoma, 2259 glaucoma) from 1725 participants.	PointNet; dynamic graph CNN (DGCNN).	Identifies critical 3D structural features of the ONH for glaucoma diagnosis.	DGCNN: AUC 0.97; PointNet: AUC 0.95.
Wu et al. [31]	2021	Cross-sectional	Spectralis spectral-domain OCT.	470 eyes (224 healthy, 246 glaucoma).	Conditional inference trees (CIT); logistic model tree (LMT); C5.0 decision tree; random forest (RF); extreme gradient boosting (XGBoost).	Glaucoma diagnosis (discriminating normal from glaucomatous eyes and distinguishing between early, moderate, and severe glaucoma from normal conditions).	Best performer: random forest Accuracy (Mean): 0.8818 Sensitivity (Mean): 0.9166 Specificity (Mean): 0.8507 AUC (Mean): 0.9459
Ashtari-Majlan et al. [32]	2025	Retrospective	3D OCT images.	1110 scans (from 624 patients; 263 healthy, 847 glaucoma).	Spatial-aware transformer–gated recurrent unit (GRU) framework.	To enable early detection of optic nerve structural damage in glaucoma patients.	AUC: 0.942; F1 score: 93.01%; Accuracy: 89.19%; Sensitivity: 91.83%; Specificity: 79.67%.
Li et al. [14]	2023	Prospective, cross-sectional study	Peripapillary RNFL thickness from OCT images.	514 participants (257 glaucoma; 257 controls).	Logistic regression (LR), support vector machines (SVM), random forests (RF), gradient boosting (GB); models combined using Soft Voting Ensembling (SVE).	To classify eyes as glaucomatous or normal.	AUC: 0.96.
De Jesus et al. [35]	2020	Retrospective	OCTA imaging, averaged circumpapillary RNFL thickness.	121 participants (39 glaucoma; 82 healthy).	Support vector machine (SVM), random forest (RF), and gradient boosting (xGB).	Classifying and staging glaucomatous vascular damage.	Healthy vs. glaucoma (AUROC): SVM: 0.89, RF: 0.86, xGB: 0.85, RNFL: 0.85.
Bowd et al. [36]	2022	Cross-sectional comparison of diagnostic approaches	OCTA (en face vessel density images) and SD-OCT (RNFL thickness).	405 eyes of 265 participants (130 eyes of 80 healthy individuals, 275 eyes of 185 glaucoma patients).	Convolutional neural network (CNN), specifically VGG16 (fine-tuned). Gradient-boosting classifier (GBC).	Classifying healthy and glaucomatous eyes.	AUPRC: 0.97.
Lee et al. [37]	2023	Retrospective	Macular OCTA and OCT images.	260 eyes (203 eyes with highly myopic glaucoma, 57 eyes with healthy high myopia).	Transformer.	Distinguishing highly myopic glaucoma eyes from healthy high myopia eyes.	AUC 0.946

**Table 3:** Summary of AI applications in molecular and omics data analysis for glaucoma diagnosis and detection.

Study	Year	Study Design	Data Type	Data Size	AI Type	Task	Performance
Dai et al. [38]	2022	Retrospective	Transcriptome sequencing data.	GEO database (GSE9944 and GSE2378).	Logistic regression (LR), random forest (RF), and lasso regression (LASSO).	Identify diagnostic biomarkers of glaucoma (specifically ENO2) based on gene expression; construct diagnostic models and screen diagnostic markers for glaucoma diagnosis.	1. Machine learning models (LR-RF, LASSO) identified key gene biomarkers (core 3: NAMPT, ADH1C, and ENO2) for glaucoma diagnosis. 2. Potential therapeutic compounds targeting ENO2 were explored via molecular docking.
Han et al. [39]	2021	Retrospective study with multi-dataset and cross-ancestry validation (using UK Biobank and CLSA data)	Retinal fundus images.	UKB images: 175,770 from 85,736 participants; CLSA images: 106,330 from 29,635 participants.	Convolutional neural network (CNN).	1. Automated AI labeling (grading) of optic nerve head parameters: Vertical cup-to-disc ratio (VCDR) and Vertical disc diameter (VDD) from retinal fundus images. 2. Enabling large-scale cross-ancestry epidemiological studies and genetic discovery (GWAS) for ONH parameters.	1. Pearson’s correlation (AI vs. clinician): VCDR 0.81 (UKB), 0.84 (CLSA); VDD 0.84 (UKB), 0.88 (CLSA). 2. Increased heritability estimates: 50% for VCDR/VDD. Identified >200 GWAS loci for VCDR/VDD.
Alipanahi et al. [40]	2021	Retrospective	Color fundus photographs.	65,680 patients.	Machine learning model.	1. To predict glaucomatous optic nerve head features from color fundus photographs. 2. Use ML-based VCDR predictions to improve genomic discovery (GWAS) for VCDR and polygenic prediction for VCDR and POAG	1. Identified 299 independent genome-wide significant (GWS) hits in 156 loci in ML-based VCDR GWAS. 2. Replicated 62 out of 65 GWS loci from the previous manual VCDR GWAS, and identified 93 novel loci.
Sergounitis et al. [41]	2024	Retrospective	Retinal OCT images.	31,135 UK Biobank participants (left eye thickness maps).	Autoencoder; U-Net.	1. Autoencoder-based phenotyping (feature extraction/representation) of retinal OCT images. 2. Highlighting genetic loci influencing retinal morphology and providing informative biomarkers.	Autoencoder phenotyping of OCT images enabled identification of 118 significant genetic loci (41 replicated).

**Table 4:** Summary of key studies applying AI models to OCT-based glaucoma progression prediction.

Study	Year	Study Design	Data Type	Data Size	AI Type	Task	Performance
Mandal et al. [46]	2024	Retrospective longitudinal study	Peripapillary OCT circle scans and sequences of OCT B-scans.	8785 follow-up sequences of 5 consecutive OCT tests from 3253 eyes.	Convolutional neural networks (CNN)-long short-term memory (LSTM) model.	1. Distinguishing glaucoma progression from age-related changes in OCT scans. 2. Classifying whether sequences of OCT B-scans showed glaucoma progression.	AUROC: 0.894; Hit ratio (at 95% specificity): 0.498.
Hou et al. [47]	2023	Retrospective longitudinal cohort study	Longitudinal OCT data.	8785 follow-up sequences of 5 consecutive OCT tests from 3253 eyes.	Gated transformer network.	To predict visual field worsening from longitudinal optical coherence tomography data.	AUC: 0.97.
Hassan et al. [48]	2020	Retrospective longitudinal study	Longitudinal macular OCT images.	109 eyes.	Conditional generative. adversarial network (GAN).	Predict glaucoma progression over time by reconstructing future macular cross-sectional OCT images.	1. Predicting from 3 prior visits: Average Structural Similarity Index Measure (SSIM) = 0.8325. 2. Predicting from 2 prior visits: Average SSIM = 0.8336.
Lazaridis et al. [49]	2021	Retrospective study	Time-domain OCT (TDOCT) and spectral-domain OCT (SDOCT).	4902 TDOCT and 1789 SDOCT (RAPID study).	Generative adversarial networks (CycleGANs).	To improve the statistical power of glaucoma clinical trials utilizing TDOCT by converting TDOCT to synthesized SDOCT and obtaining improved RNFL segmentation.	1. 95% Limits of Agreement (LOA) improved from [26.64, −22.95] μm (original TDOCT) to [6.57, −5.79] μm (synthesized SDOCT). 2. Required sample size falls from 7356 (original TDOCT) to 578 (synthesized SDOCT).
Hussain et al. [50]	2023	Retrospective longitudinal study	OCT images, visual field (VF) values, demographic data, and clinical data.	86 glaucoma patients with five visits over 12 months.	Combining a convolutional neural network (CNN) and a long short-term memory (LSTM) network, guided by a generative adversarial network (GAN).	To predict VF changes 12 months after the first visit.	Best AUC: 0.83 (for predicting progression 6 months early).
Bowd et al. [51]	2021	Prospective longitudinal cohort study	3D OCT images and segmented RNFL thickness maps.	44 progressing glaucoma eyes, 189 nonprogressing glaucoma eyes, 109 healthy eyes.	Deep-learning autoencoders (DL-AEs).	To compare individualized OCT RNFL-based region of interest (ROI) maps to conventional global circumpapillary RNFL (cpRNFL) thickness for detecting progression.	Sensitivity for detecting change in progressing eyes: DL-AE ROIs = 0.90 vs. cpRNFL annulus = 0.63.

**Table 5:** Summary of key studies on AI-driven visual field-based glaucoma progression prediction.

Study	Year	Study Design	Data Type	Data Size	AI Type	Task	Performance
Mohammadzadeh et al. [52]	2024	Retrospective longitudinal study	Serial optic disc photographs and baseline retinal nerve fiber layer (RNFL) thickness from OCT, serial visual field.	3079 eyes (1765 patients).	Siamese Neural Network with ResNet-152 backbone pretrained on ImageNet.	To predict visual field progression based on baseline and longitudinal structural measurements.	Predicting fast progression (MD rate < 1.0 dB/year): AUC: 0.911.
Mohammadzadeh et al. [53]	2024	Prospective longitudinal cohort study	Longitudinal pairs of optic disc photographs, visual field, and RNFL thickness maps from SD-OCT.	3919 eyes (2259 patients).	Twin-neural network with ResNet50-backbone.	To predict visual field progression based on longitudinal pairs of optic disc photographs.	Predicting fast progression (MD rate < −1.0 dB/year): AUC: 0.926; Sensitivity: 100%; Specificity: 80.0%; Accuracy = 80.4%.
Yoesef et al. [54]	2020	Retrospective, cross-sectional, longitudinal cohort study	Visual fields (VFs) from standard automated perimetry (SAP).	13,231 VFs from the most recent visit of each patient (from a total of 31,591 VFs on 8077 subjects).	Artificial intelligence (AI) dashboard enabled. Pipeline included principal component analysis (PCA), manifold learning, and unsupervised clustering. Density-based clustering and archetypal analysis were used for annotation.	To develop an AI dashboard for monitoring glaucomatous functional loss.	Dashboard identified 32 nonoverlapping clusters corresponding to global functional severity, extent of VF loss, and characteristic local patterns. Specificity for detecting “likely nonprogression”: 94% Sensitivity for detecting “likely progression”: 77%.
Dixit et al. [55]	2021	Retrospective study	Longitudinal visual field (VF) data and clinical data. Clinical data include baseline cup-to-disc ratio, central corneal thickness, and intraocular pressure (IOP).	11,242 eyes.	Convolutional long short-term memory (LSTM) neural network.	To detect when glaucoma progression is occurring based on longitudinal VF and clinical data.	Accuracy: 91% to 93%; AUC: 0.89–0.93.
Saeeedi et al. [56]	2021	Retrospective study	Visual fields.	90,713 visual fields from 13,156 eyes.	Logistic regression, random forest, extreme gradient boosting, support vector classifier, convolutional neural network, fully connected neural network.	To classify each eye as progressing or stable.	Accuracy: 87% to 91%; Sensitivity: 0.83–0.88; Specificity: 0.92–0.96; Precision: 0.90–0.95; NPV: 0.84–0.88; F1 score: 0.87–0.91.
Shuldiner et al. [57]	2021	Retrospective study	Visual fields.	22,925 initial VFs from 14,217 patients.	Support vector machine (SVM), random forest (RF), naïve Bayes, logistic regression, fully connected neural network, and hybrid model.	Predicting future rapid glaucoma progression based on an initial visual field test.	Support vector machine model AUC: 0.72.
Sabharwal et al. [58]	2023	Retrospective study	Visual field testing data and clinician assessment from health records.	5099 patients (8705 eyes).	Deep learning model.	Detection of visual field worsening in glaucoma patients.	AUC: 0.94.
Chen et al. [59]	2024	Retrospective study	Visual fields.	2430 eyes of 1283 patients.	Multi-label transformer-based network (MTN).	Prediction of focal visual field progression in six VF regions.	Macro-average AUCs for detecting focal VF progression (5+ VFs): 0.884.

**Table 6:** Summary of key studies on AI-driven prediction of glaucoma surgical outcomes.

Study	Year	Study Design	Data Type	Data Size	AI Type	Task	Performance
Banna et al. [63]	2022	Retrospective cohort study	Preoperative systemic data, demographic data, and ocular data.	230 trabeculectomy surgeries performed on 184 patients.	Random forest (RF), support vector machine (SVM), artificial neural networks, and multivariable logistic regression.	To predict the complete success of trabeculectomy surgery at 1 year.	Top performing model (random forest): Accuracy: 0.68; AUC: 0.74.
Barry et al. [64]	2024	Retrospective study	Structured data from electronic health records (EHRs), including demographics, prior diagnosis and procedure codes, medications, and eye exam findings (intraocular pressure, visual acuity, central corneal thickness, and refraction spherical equivalent).	2398 glaucoma surgeries of 1571 patients.	Benchmarked classical ML classifiers (decision trees, random forest, XGBoost, penalized logistic regression, multilayer perceptron, k-nearest neighbors, Gaussian naive Bayes, linear discriminant analysis, support vector machines) and feedforward deep learning models. Random forest and a neural network performed best for overall surgical failure.	To predict glaucoma surgical outcomes, including postoperative intraocular pressure, use of ocular antihypertensive medications, and need for repeat surgery. Predict overall surgical failure (composite criteria) and individual failure criteria (IOP failure, medication failure, and follow-up surgery failure).	Random forest: Accuracy 75.5%; AUROC 76.7%; F1 0.850; Sensitivity 0.955; Specificity 0.223; Precision 0.765; NPV 0.660. Neural network (with embedding): Accuracy 75.5%; AUROC 76.6%; F1 0.837; Sensitivity 0.870; Specificity 0.452; Precision 0.807; NPV 0.570.
Lin et al. [65]	2024	Retrospective study	Multimodal data: structured EHR data (demographics, diagnoses, medications, eye exam findings, etc.) and free-text operative notes (intraoperative information, findings, techniques, complications, etc.).	1540 eyes from 1326 patients who underwent primary trabeculectomies.	Multimodal deep learning models (neural networks). Combines structured data input with processed free-text operative notes. Text processing models evaluated: transformer encoder blocks, LSTM units, and pretrained Bio-Clinical BERT.	To predict multiclass surgical outcomes for glaucoma trabeculectomy surgery at 1 year.	Surgical success: Precision 0.884 (highest precision), F1 score 0.775.
Agnifili et al. [66]	2023	Prospective cohort study	Preoperative clinical parameters: ocular surface clinical tests (OSCTs), surgical site-related biometric parameters (SSPs), and conjunctival vascularization. AS-OCT measurements: conjunctival epithelial and stromal (CET, CST) thickness and reflectivity (ECR, SCR). Baseline intraocular pressure (IOP).	102 glaucomatous patients undergoing filtration surgery.	Classification tree.	To predict the filtration surgery outcome (success vs. failure).	AUC: 0.784.
Mastropasqua et al. [67]	2023	Retrospective, cross-sectional study	Slit-lamp images of filtration blebs (FBs) after trabeculectomy.	119 post-trabeculectomy filtration blebs images.	Deep learning model; artificial intelligence classification algorithm.	To distinguish functioning from failed filtration blebs using deep learning on slit-lamp images.	Accuracy: 74%; Overall sensitivity: 74; Overall specificity: 87%; AUROC: 0.8; AUPRC: 0.74.

## Data Availability

No new data were created or analyzed in this study. Data sharing is not applicable to this article.
